# Expert consensus on passive heating interventions to promote skeletal muscle strength and mass: a delphi study

**DOI:** 10.1007/s00421-026-06295-6

**Published:** 2026-06-15

**Authors:** Elisa Lemos, Ian B. Stewart, Patrick Rodrigues, Shawnda A. Morrison, Lee Wharton, Christopher J. Tyler, Stephen S. Cheung, Sebastian Racinais, Ralph J. F. H. Gordon, Alex Lloyd, Julien D. Périard, Katsumasa Goto, Gabriel S. Trajano, Geoffrey M. Minett

**Affiliations:** 1 School of Exercise and Nutrition Sciences, Faculty of Health, Queensland University of Technology, Brisbane, Australia; 2 School of Sport and Human Movement, University of Waikato, Hamilton, New Zealand; 3 Faculty of Sport, University of Ljubljana, Ljubljana, Slovenia; 4 School of Life and Health Sciences, University of Roehampton, Danebury Avenue, London, England, UK; 5 Environmental Ergonomics Laboratory, Department of Kinesiology, Brock University, St. Catharines, ON Canada; 6 Research and Scientific Support Department, Aspetar Orthopedic and Sports Medicine Hospital, Doha, Qatar; 7 Environmental Stress Unit, CREPS Montpellier Font-Romeu, Montpellier, France; 8 DMEM, Univ Montpellier, INRAE, Montpellier, France; 9 Skin Sensing Research Group, School of Health Sciences, University of Southampton, Southampton, UK; 10 Environmental Ergonomics Research Centre, Loughborough School of Design and Creative Arts, Loughborough University, Loughborough, UK; 11 Research Institute for Sport and Exercise (UCRISE), University of Canberra, Bruce, Australia; 12 Department of Physiology, Graduate School of Health Sciences, Toyohashi SOZO University, Toyohashi, Aichi Japan

**Keywords:** Thermal therapy, Heat stress, Thermal strain, Hot-water immersion, Skeletal muscle adaptation

## Abstract

**Purpose:**

Passive heating has emerged as a potential non‑exercise method to support muscle strength and mass, but its application is limited by inconsistent protocols and unclear dosage and safety guidelines. This study used a Delphi methodology to establish expert consensus on key parameters for passive heating interventions targeting neuromuscular and muscle morphology outcomes.

**Methods:**

An international panel of academic experts (≥ 3 peer‑reviewed publications in passive heating and neuromuscular function) completed a two‑round Delphi process. Round 1 gathered open‑ended responses on passive heating strategies, intervention targets, and safety. In Round 2, experts ranked the importance of generated statements. Consensus was defined as ≥ 70% ranking a statement as either first or second in importance.

**Results:**

Eleven experts completed Round 1 and ten in Round 2. From 177 qualitative responses, 130 statements were evaluated, with 11 reaching consensus (80–100% agreement; interquartile range = 0–1). Hot‑water immersion was prioritised as the most optimal passive heating modality. Experts agreed on targeting muscle temperatures of 39–40 °C, ~ 60-minute session durations, and once‑daily exposure as key intervention parameters. Strong consensus was also reached for monitoring core temperature and cardiovascular strain (e.g., heart rate, blood pressure) to ensure participant safety. No consensus was reached on a single target population or the total intervention duration.

**Conclusion:**

This Delphi study provides a consensus‑based framework outlining key parameters and safety considerations for passive heating interventions. These findings do not establish efficacy but identify priority variables to inform future experimental research and cautious translation into applied and clinical contexts.

## Introduction

The force-generating capacity of skeletal muscle is critical for activities of daily living, independence, and overall health (Fitts 2003; Westcott 2012). Although resistance training effectively improves muscle strength and mass, only 10–30% of adults meet current recommendations (Sousa et al. 2016). Beyond long‑term adherence challenges, many individuals also experience periods in which resistance training is impractical or contraindicated. Such situations may include acute or prolonged limb immobilisation (e.g. fracture or post‑surgical recovery), early rehabilitation following musculoskeletal injury, advanced frailty in older adults, severe joint pain or inflammatory conditions, or medical states where high‑load mechanical stress is temporarily contraindicated. Critically, muscle strength declines with ageing, immobilisation, or prolonged disuse, contributing to frailty, increased fall risk, and chronic disease burden (Brazaitis et al. 2019; Kalyani et al. 2014; Metter et al. 2002). Together, these limitations underscore the need for alternative or adjunct strategies to preserve muscle function in populations who are unable or unwilling to engage in traditional resistance training.

Passive heating has shown potential to stimulate several signalling pathways and morphological responses that overlap with those observed following resistance training (Kim et al. 2020a; Rodrigues et al. 2020). Repeated heat exposure may lead to capillary growth, hypertrophy, and increased mitochondrial content and function (Goto et al. 2011; Kim et al. 2020a). These adaptations are achieved through anabolic signalling, which involves upregulating heat shock proteins (HSPs) (Naito et al. 2012; Ogura et al. 2007), calcineurin expression (Wu et al. 2000), activation of the protein kinase B (ATK)/rapamycin complex 1 (mTORC1) pathway (Ihsan et al. 2020), and NfKB pathway inhibition (Dablainville et al. 2025; Labidi et al. 2024). Studies have demonstrated that heat stress can protect against reductions in human skeletal muscle protein synthesis during immobilisation (Hafen et al. 2019; Labidi et al. 2024), suggesting that passive heating via strategies such as hot baths at home might be a viable way to maintain muscle mass and function.

Despite the promise of passive heating to enhance muscle strength and muscle mass, the mechanisms and practical implementation of these interventions remain poorly defined. Existing studies employ heterogeneous heating modalities, temperatures, exposure durations, and intervention lengths, making it difficult to identify optimal protocols or compare outcomes across studies (Rodrigues et al. 2020). As a result, clinicians and practitioners lack evidence-informed guidance to translate passive heating into safe and effective practice. While not all cited studies directly assess muscle strength or muscle mass, passive heating has been explored as a therapeutic strategy in populations such as older adults (Armstrong and Kenney 1993; Brazaitis et al. 2019; Fuchs et al. 2025), sedentary overweight individuals (Hoekstra et al. 2018), and those with soft‑tissue injury or limb immobilisation (Dablainville et al. 2025; Hafen et al. 2019; Labidi et al. 2024), primarily for thermophysiological, cardiovascular, or metabolic outcomes with potential indirect relevance to musculoskeletal health. Importantly, neuromuscular and muscle morphology outcomes impose distinct physiological and safety requirements (e.g., tissue‑level thermal strain, exposure duration, and monitoring thresholds), which limit the direct applicability of conclusions drawn from broader passive heating research and from isolated neuromuscular studies using heterogeneous protocols.

Where empirical evidence is limited not only in volume but also in methodological consistency and dose–response clarity, the Delphi method provides a structured approach to prioritising intervention variables and safety considerations based on expert judgement (Hasson et al. 2000; Jünger et al. 2017). In this context, the Delphi consensus is not intended to define optimal prescriptions but rather to identify and prioritise key parameters warranting systematic experimental investigation in emerging or heterogeneous research fields (Rowe and Wright 1999). Unlike narrative or systematic reviews, which primarily summarise existing data, Delphi studies are specifically designed to structure expert judgement to prioritise variables and safety considerations when definitive experimental evidence is lacking. As such, this approach is particularly well suited to the emerging field of passive heating, where clear dose–response relationships and implementation frameworks have yet to be established, and where expert prioritisation can guide future experimental work.

The purpose of this study was to use a Delphi methodology to identify and prioritise key intervention parameters and safety considerations considered essential by experts when passive heating is used with the intention of supporting muscle strength and muscle mass. Specifically, the study sought agreement on heating modality, target temperatures, session duration, frequency, intervention length, and essential safety considerations. By integrating expert consensus across physiology, rehabilitation, and applied practice, this work provides an evidence-informed framework to guide future experimental research and early implementation efforts in populations at risk of neuromuscular decline. Rather than providing empirical recommendations, this consensus‑based framework is intended to inform the design, prioritisation, and harmonisation of future experimental studies and to identify minimum safety considerations for early translational work.

## Methods

### Participants

Participant selection is the most crucial aspect of Delphi studies (Green et al. 1999). Consequently, a purposive sampling method was used to recruit a range of academic experts. Experts were required to have at least three peer‑reviewed publications directly examining passive heating interventions and neuromuscular, muscular, or thermophysiological outcomes in humans, irrespective of authorship position (i.e., first, last, or co‑author). The systematic review and meta‑analysis by Rodrigues et al. (2020), which examined the effects of passive heating on neuromuscular function, served as a foundational source for identifying researchers with established expertise in this field. To ensure comprehensive and up‑to‑date identification of potential Delphi panel members, a further literature search was then conducted in PubMed, Web of Science, Scopus, CINAHL, EMBASE, Cochrane, and SPORTDiscus using the same eligibility criteria as Rodrigues et al. (2020), which included original peer-reviewed studies involving passive heating interventions in humans, published in English, and addressing neuromuscular, cardiovascular, or thermoregulator outcomes. This approach enabled cross‑referencing and updating the pool of eligible experts based on recent, relevant publications. Participants were invited via email, with no restrictions on country of origin or publication date. No financial compensation was offered.

A total of 11 experts completed Round 1, and 10 experts completed Round 2 of the Delphi process. A panel of 10–18 experts is considered sufficient in Delphi studies when participants possess high‑level domain expertise (Hasson et al. 2000; Kleynen et al. 2014). Accordingly, the panel size in the present study is consistent with established methodological guidance.

Given the specialised nature of passive heating research, some overlap in publication histories and collaborative networks among panel members was unavoidable. To minimise potential bias arising from shared intellectual frameworks, all responses were anonymised, contributions were blinded between participants, and consensus was derived from independent forced‑ranking rather than group discussion, consistent with best‑practice Delphi recommendations (Hasson et al. 2000; Kleynen et al. 2014).

### Research design

This study employed a two-round Delphi design to establish expert consensus on passive heating intervention parameters for neuromuscular and muscle morphology outcomes. The study was conducted and reported in accordance with established recommendations for Delphi research, including anonymised participation, controlled feedback, and predefined consensus criteria (Hasson et al. 2000; Jünger et al. 2017).

To enhance interpretability and comparability, the domains explored in this Delphi study align conceptually with the Frequency, Intensity, Time, and Type (FITT) principles commonly applied in exercise prescription (Rodrigues et al. 2025).

Expert consensus was obtained via a de-identified online questionnaire administered using the Qualtrics survey platform. The questionnaire served two purposes. First, demographic, professional, and academic background information (e.g., professional role, academic expertise, h-index, and publication record) was collected to characterise the Delphi panel’s expertise and credibility. Self‑reported categorical h‑index and publication record information were used descriptively to characterise panel expertise and were not verified against bibliometric databases. Second, experts were asked to provide their opinions and rate their level of agreement on variables related to passive heating strategies and neuromuscular outcomes. To minimise potential bias associated with group conformity or reputational dominance, participants were blinded to the contributions of other panel members throughout the process.

The Delphi process was conducted using Qualtrics CoreXM (Qualtrics.com), an Internet-based software program administered through Queensland University of Technology (QUT). Survey invitations were distributed via email with a live link to the data collection tool (QUT Ethics Approval Number 4112).

Although traditional Delphi studies may involve up to four rounds, consensus is often achieved within two rounds when expert panels demonstrate high domain expertise (Jünger et al. 2017). A two-round Delphi design was deemed appropriate given the focused scope of the research questions, the high level of domain expertise among panel members, and the structured opportunity for response refinement through controlled feedback between rounds. Controlled feedback consisted of anonymised summaries of group responses provided to participants between rounds to inform subsequent ratings (Rowe and Wright 1999). The Delphi process was terminated after two rounds because predefined consensus thresholds were achieved and response stability was observed.

Items in each round were organised into three categories: passive heating strategies, intervention targets, and intervention safety controls. Round 1 consisted of open-ended questions designed to generate statements based on expert opinion. In Round 2, experts ranked the five most important statements for each question in order of importance (1 = most important; 5 = least important). This ranking approach was used to prioritise relative importance and to reduce central-tendency bias. Statements not ranked among the top five were retained for descriptive purposes but were not considered to have reached consensus.

### Development of the questionnaire

The initial Delphi questionnaire was developed and reviewed by the core research team (ECL, IS, LW, GM, GT) and comprised 18 main questions. Question development was informed by the existing literature on passive heating and neuromuscular adaptations, prior experimental protocols, and the collective expertise of the research team. Members of the core panel were not included as Delphi respondents. The questionnaire addressed three topic areas: (i) passive heating strategies (questions 1–11); (ii) intervention targets (questions 12–15); and (iii) intervention safety controls (questions 16–18) (see Appendix 1).

### Round 1

The Round 1 questionnaire was divided into two parts. Part 1 collected participant demographic and professional information, including primary country of work, professional background, h‑index, and the number of peer‑reviewed publications on passive heating and neuromuscular function. These self-report data were collected to characterise the expertise of the Delphi panel.

Part 2 aimed to generate statements on passive heating methods and dosage variables, based on expert opinion. Eighteen open‑ended questions addressed key protocol dimensions, including heating modality, thermal intensity, session duration, intervention frequency, and total intervention length required to promote neuromuscular and muscle morphology adaptations. Experts were also asked to identify and explain individual characteristics and contextual factors that may influence the prescription, tolerability, or effectiveness of passive heating interventions.

Responses from Round 1 were collected over a one‑month period, with reminder emails that included information regarding voluntary participation and withdrawal. Controlled feedback was provided after the round, consisting of anonymised summaries of group responses (Rowe and Wright 1999). Responses were inductively clustered into thematic statements but were not quantitatively analysed or interpreted within the feedback reports.

### Round 2

Round 2 aimed to evaluate consensus on statements related to passive heating strategies, intervention targets, and intervention safety controls identified in Round 1. Experts ranked the five most important statements for each question (1 = most important; 5 = least important). This forced‑ranking approach was used to prioritise relative importance and reduce central tendency bias.

### Data analysis

Data were exported from Qualtrics into Microsoft Excel and subsequently analysed using SPSS (IBM Corp., version 31). Consensus was defined a priori as ≥ 70% of experts ranking a statement as either first or second in importance, consistent with previous Delphi studies (Hoekstra et al. 2018; Jünger et al. 2017; Kleynen et al. 2014). Consensus strength was quantified using the interquartile range (IQR) of experts’ responses on a five‑point ordinal rating scale (1–5). Consistent with established Delphi methodology, lower IQR values indicated greater agreement, with IQR = 0 reflecting strong consensus, IQR = 1 moderate consensus, and IQR ≥ 2 indicating dispersed opinions.

## Results

### Study flow and panel participation

Thirteen experts were invited to participate in Round 1, of whom 11 completed the questionnaire (response rate: 84.6%). Ten experts completed Round 2. For selected items in Round 2, response numbers varied due to item‑specific non‑responses (*n* = 9 for questions 3, 4, 6, and 10; *n* = 8 for question 7).

At the time of the study, participants were based in Australia (*n* = 2), Canada (*n* = 1), Japan (*n* = 1), Singapore (*n* = 1), Slovenia (*n* = 1), Qatar (*n* = 1), and the United Kingdom (*n* = 3). Panel members reported a mean of 13.6 ± 7.3 years of experience working in relevant academic or applied fields. The participants’ scholarly output and performance metrics included h‑indices of < 10 (*n* = 3), 11–20 (*n* = 4), 21–30 (*n* = 3), and > 40 (*n* = 1), and publication counts of < 50 (*n* = 5), 51–100 (*n* = 4), and 101–200 (*n* = 2) peer‑reviewed articles.

### Round 1: statement generation

In Round 1, experts provided 177 qualitative responses in the form of short narratives and discrete statements addressing passive heating strategies, intervention targets, and safety considerations. Responses were analysed using inductive content analysis, in which open‑ended responses were independently reviewed, conceptually similar ideas were grouped, and overlapping responses were consolidated without a predefined coding framework. This process resulted in 130 unique statements. These statements were organised into three predefined thematic categories: (i) passive heating strategies, (ii) intervention targets, and (iii) intervention safety controls. All 130 statements were advanced to Round 2 for quantitative evaluation. A complete list of statements generated in Round 1 is provided in Tables 1, 2 and 3.

### Round 2: consensus outcomes

In Round 2, experts ranked the relative importance of statements within each thematic category. Of the 130 statements evaluated, consensus, defined as ≥ 70% of experts ranking a statement as either first or second in importance, was achieved for 11 statements. Consensus, therefore, reflects relative prioritisation among expert‑generated statements, rather than an absolute judgement of their adequacy or empirical superiority. A summary of all statements reaching consensus, including agreement percentages, medians, and IQRs, is presented in Table 4. Unless otherwise stated, percentages are based on the number of respondents for each item.

**Table 1:** List of statements of theme 1: passive heating strategies

Subtheme		Statements
1.	Reasons why water immersion is the most efficient	1.1. The most effective heat transfer and thermal conductivity provide a more uniform heating
		1.2. The most accessible for the broadest range of individuals
		1.3. The ability to target part- or whole-body regions
		1.4. Heating can occur quickly and be controlled, also increasing pressure
		1.5. Prolonged warming effect that promotes a sustained increase in core and muscle temperature
		1.6. It seems to benefit the clinical population, as it is not as uncomfortable as other methods
		1.7. It is efficient, applicable, affordable, and simple to calibrate and maintain
2.	Influence that body surface area exposed to the hot-water immersion has on neuromuscular and/or muscle mass responses	2.1. It is associated with muscle temperature, which impact on neuromuscular and muscle mass responses
		2.2. It influences the heat storage and distribution rate to the body, which may benefit muscle mass
		2.3. It influences the core temperature, which may impair the neuromuscular responses independent of muscle temperature
		2.4. Whole-body heating affects core temperature and, thus, oxidative, immune, and cardiovascular stress/strain
		2.5. Local heating is associated with neuromuscular adaptation
		2.6. It may affect the tolerability to the heat, which may negatively impact the benefits expected
3.	Variables to control the heat stress in a hot-water immersion intervention	3.1. Muscle temperature
		3.2. Core temperature
		3.3. Water temperature
		3.4. Humidity
		3.5. Ambient temperature
		3.6. Cardiovascular response (e.g., blood pressure, heart rate)
		3.7. Weighed body temperature
		3.8. Skin temperature
		3.9. Thermal comfort and thermal sensation scales
4.	Variables for monitoring participants’ safety	4.1. Core temperature (rectal or oesophageal)
		4.2. Cardiovascular strain (blood pressure, heart rate)
		4.3. Thermal sensation
		4.4. Thermal comfort
		4.5. Symptoms and signs: skin colour, speech, nausea, syncope, volitional exhaustion, request for cessation, etc
		4.6. Temperature of the apparatus
		4.7. Cognitive function/attentiveness/ability to focus forms
		4.8. Skin temperature
		4.9. Subjective ratings
		4.10. Sweating rate
		4.11. Pre-testing is important to avoid low-temperature burns
5.	Strategies to be considered when designing a protocol to promote neuromuscular and muscle morphology benefits	5.1. Individual factors (age, sex, body composition)
		5.2. Physical fitness (fitness level, training history)
		5.3. Health status (e.g., clinical conditions, clinical needs, immobilisation, and underlying disease)
		5.4. The mode of heating (temperature used, equipment available)
		5.5. The specific purpose of the intervention (atrophy attenuation vs. maximising or maintaining hypertrophy/strength)
		5.6. Core temperature response to the heat
		5.7. Accessibility
		5.8. Adherence
		5.9. Supervision
		5.10. Avoiding low-temperature burns
6.	Intensity	6.1. Water temperature at 39 °C
		6.2. Water temperature at 40 °C
		6.3. Water temperature at 41 °C
		6.4. Water temperature at 42 °C
		6.5. Muscle temperature between 39 and 40 °C
		6.6. Elevation of 1 to 1.5 °C on core temperature
		6.7. Elevation of 1 to 1.5 °C on muscle temperature
7.	Duration	7.1. 20–30 min
		7.2. 45 min
		7.3. 60 min
		7.4. 90 min
		7.5. 120 min
8.	Frequency of passive heating sessions (per week)	8.1. Three sessions per week
		8.2. Four sessions per week
		8.3. Five session per week
		8.4. Six sessions per week
		8.5. Seven sessions per week
9.	Passive heating schedule	9.1. Once a day
		9.2. Multiple sessions daily
		9.3. Less regularly

### Passive heating strategies

A consensus was reached for several statements related to passive heating strategies. Hot‑water immersion was identified as the most effective passive heating modality for influencing muscle strength and mass-related outcomes, with 80% of experts ranking it among the two most important methods (IQR = 0). Regarding heating intensity, consensus was reached to target a muscle temperature between 39 °C and 40 °C (80% agreement; IQR = 1).

**Table 2:** List of statements generated for theme 2: intervention target

Subtheme		Statements
10.	Who would most benefit from passive heating therapies	10.1. Untrained individuals
		10.2. Elderly
		10.3. Immobilisation (e.g., due to injury)
		10.4. Athletes in rehab
		10.5. Clinical population
		10.6. People with locomotive problems
		10.7. Anyone can benefit
		10.8. Muscle atrophy or weakness aligned with conditions like Parkin’s disease, amyotrophic lateral sclerosis (ALS), Guillain-Barre syndrome, multiple sclerosis, muscular dystrophy, and neuropathy
		10.9. Those who have suffered from previous vasodilatory, vasoconstriction issues (i.e., amputees)
		10.10. Returning from or during bed hospitalisation
11.	Neuromuscular adaptation to passive heating	11.1. Improved peak twitch torque
		11.2. Improved voluntary muscle force or torque
		11.3. Improved half-relaxation time (faster)
		11.4. Improved rate of torque development
		11.5. Improved calcium reuptake
		11.6. Improved electromechanical delay
12.	Musculoskeletal adaptations to passive heating	12.1. Muscle hypertrophy
		12.2. Changes in mitochondrial function and/or content
		12.3. Increased capillarisation
		12.4. Decreased rate of muscle atrophy
		12.5. Heat shock protein expression

**Table 3:** List of statements generated for theme 3: intervention safety controls

Subtheme		Statement
13.	Factors that could interfere with passive heating protocol, potentially affecting neuromuscular and muscle morphology benefits	13.1. Electric fans and cold-water consumption help with heat stress tolerance and comfort
		13.2. Hydration status by helping with thermoregulatory responses during the session
		13.3. Inter-individual factors (e.g., trained vs. non-trained, diet, sleep)
		13.4. Many of the factors listed can improve the potential for gains
		13.5. Cooling strategies can negatively affect the benefits of passive heating
		13.6. Pharmacological factors may impair the benefits of passive heating
		13.7. Cooling and heating can co-exist, provided they are done separately and do not interfere with each other’s primary physiological response
		13.8. All listed in the question will help with participant comfort
		13.9. The phase of the menstrual cycle
		13.10. Substrate availability (e.g., weight-cutting practices generally have athletes in a nutrient-poor state)
		13.11. Jet lag (i.e., circadian rhythm misalignment) can affect the core temperature response
		13.12. High altitude/hypoxia (people acclimated to high altitude)
		13.13. Acclimation/acclimatisation status
14.	Factors that should be considered when applying heat stress safely	14.1. Monitor participant signs and symptoms during the session
		14.2. Having a research/clinician supervising the session
		14.3. Monitoring equipment
		14.4. Familiarisation with heat stress
		14.5. Never leave the patient alone
		14.6. Monitoring daily heat stress, external exercise, and heat exposure
		14.7. Emergency care when working with vulnerable people
		14.8. Screening for contraindications
		14.9. Monitor physiological responses
		14.10. Pre-testing to avoid low-temperature burns
15.	Important aspects to consider when designing a passive heating protocol	15.1. Participant tolerance to heat (i.e., increase the intensity of the heat in each session gradually)
		15.2. Taking into consideration the purpose of passive heating/intervention (rehab vs. maintenance during injury recovery)
		15.3. The financial and environmental cost
		15.4. Find the lowest intensity that promotes benefits to avoid heat radiation
		15.5. The number of measurements could affect participant comfort
		15.6. Participant enjoyment

A strong consensus was also observed for session duration, with 90% of experts ranking 60 min as one of the two most important exposure durations (IQR = 0.75). Regarding session scheduling, 80% of experts ranked once‑daily passive heating as their preferred approach (IQR = 1). No consensus was reached on the total number of sessions or weeks required to elicit neuromuscular or muscle-morphology adaptations.

Regarding monitoring variables, consensus was reached on the importance of tracking core temperature during passive heating sessions (100% agreement; IQR = 0). Cardiovascular strain, assessed via heart rate and blood pressure, also reached consensus as an essential monitoring variable (88.9% agreement; IQR = 0).

**Table 4:** Consensus level, median and IQR of statements obtained from Round 2

Consensus	Consensus level (agreement in %)	Median	IQR
Passive heating strategies Efficiency of HWI (*N* = 10)			
The most effective heat transfer and thermal conductivity to provide more uniform heating	80	1.0	0.00
Intensity (*N* = 10)			
Muscle temperature between 39 °C and 40 °C	80.0	2.0	1.00
Participant safety (*N* = 9)			
Core temperature	100.0	1.0	0.00
Cardiovascular strain (blood pressure, heart rate)	88.9	2.0	0.00
Duration (*N* = 10)			
60 min	90.0	2.0	0.75
Schedule (*N* = 10)			
Once a day	80.0	1.5	1.00
Less regularly	70.0	2.0	0.75
Intervention target Factors affecting PH (*N* = 10)			
Inter-individual factors (e.g., trained vs. non-trained, diet, sleep).	70.0	2.0	1.75
Intervention safety controls			
PH safety (*N* = 10)			
Screening for contraindications	70.0	1.5	1.75
Other aspects (*N* = 9)			
Taking into consideration the purpose of passive heating (rehab vs. maintenance during injury recovery)	87.5	1.0	1.00
Participant tolerance to heat (i.e., increase gradually the intensity of the heat in each session)	87.5	2.0	1.00

### Intervention targets

No consensus was reached regarding a single population that would most benefit from passive heating interventions. Similarly, no agreement was achieved on population‑specific application strategies. However, inter‑individual factors, such as training status, diet, and sleep, were identified as important considerations influencing passive heating responses, reaching the consensus threshold with 70% agreement (IQR = 1.75).

### Intervention safety controls

Consensus was achieved for screening participants for contraindications prior to the application of passive heating interventions (70% agreement; IQR = 1.75). Additionally, experts reached consensus on the importance of considering the specific purpose of the passive heating intervention (e.g., rehabilitation vs. maintenance during injury recovery), with 87.5% agreement (IQR = 1). Participant tolerance to heat, including the need to progressively adjust heating intensity across sessions, also reached consensus (87.5% agreement; IQR = 1).

### Summary of consensus findings

Across the three thematic categories, expert consensus was achieved for 11 statements. Within the passive heating strategies domain, consensus was reached for hot‑water immersion as the preferred heating modality, a target muscle temperature of 39–40 °C, session durations of approximately 60 min, and once‑daily exposure as the preferred scheduling approach. In addition, experts achieved consensus on the importance of monitoring core temperature and cardiovascular strain (heart rate and blood pressure) during passive heating. Within the intervention target and safety domains, consensus was reached on screening participants for contraindications, considering the specific purpose of the intervention (e.g., rehabilitation vs. maintenance), accounting for participant heat tolerance through progressive exposure, and recognising inter‑individual factors (e.g., training status, diet, sleep) as influential moderators of response. Statements that did not reach the predefined consensus threshold are reported descriptively in Appendix 2–4.

## Discussion

This Delphi study established expert consensus on a limited set of priority parameters and safety considerations for passive heating interventions intended to influence factors related to muscle strength and mass. Specifically, experts identified hot‑water immersion as the preferred heating modality, prioritised achieving a muscle temperature of approximately 39–40 °C during sessions of ~ 60 min performed once daily, and emphasised the importance of monitoring core temperature and cardiovascular strain to ensure safety. In addition, consensus highlighted the need to screen for contraindications, tailor protocols to the intended application (e.g., rehabilitation vs. maintenance), and account for inter‑individual factors that influence heat tolerance and response. These findings reflect expert prioritisation rather than experimentally established dose–response relationships and provide a structured framework for interpreting existing evidence and guiding future experimental research. That several consensus outcomes align with prevailing interpretations of the literature should be viewed as reinforcing, rather than diminishing, their importance, as this convergence demonstrates agreement across heterogeneous empirical findings and independent expert judgement.

### Passive heating strategies

Traditional approaches to passively raising body temperature (e.g., thermal spring baths, Finnish sauna) have long been used to improve health (Barfield and Hodder 1987; Jackson 1990), while more recent studies have examined modalities such as electric heating pads, ultrasound, and shortwave diathermy as targeted methods to evoke thermal strain (Richey et al. 2026). In the present Delphi study, experts ranked hot‑water immersion as the most important passive heating modality (Round 1: 72.7%). This prioritisation likely reflects its capacity for effective heat transfer and thermal conductivity, providing a more uniform heating (Round 2: 80%). This expert prioritisation has since been empirically reinforced, with evidence that hot‑water immersion elevates muscle and core temperatures more effectively than other commercially available heating devices (Nasir et al. 2026), thereby scientifically validating its recognised efficacy.

Although hot‑water immersion was prioritised as a passive heating modality, the Delphi process did not establish consensus regarding the optimal extent of body or muscle mass immersion (e.g., whole‑body vs. localised immersion). Consequently, these findings should be interpreted as relating to the modality itself rather than defining the minimum spatial exposure required to elicit neuromuscular or muscle morphology adaptations. Beyond these methodological considerations, the feasibility of passive heating via hot water remains high due to its simplicity, low financial cost, and accessibility in domestic settings. However, environmental considerations, including water and energy use, warrant further attention in future implementation and translational studies. Such work could examine strategies to improve the sustainability of passive heating interventions, such as optimising session scheduling, improving heat retention, or integrating interventions within existing heating infrastructure, while preserving efficacy and safety.

Limited experimental evidence describes how dosage variables of hot-water immersion influence subsequent thermal strain and neuromuscular responses (Rodrigues et al. 2020b). Although higher temperatures, greater body surface area exposure, and longer immersion durations can increase body temperature, experts did not reach consensus that increasing these variables necessarily confers greater neuromuscular benefit. This aligns with existing evidence suggesting that the relationship between thermal dose and muscle strength and mass is non‑linear and context-dependent (Rodrigues et al. 2020b). Notably, experts differed in their views regarding how body surface area influences muscle strength and/or muscle mass responses. Consequently, recommendations for whole-body passive heating should balance water temperature and exposure duration to maintain user safety and comfort.

While part‑body approaches may permit higher water temperatures, they typically result in smaller increases in core temperature. However, localised heating may be perceived as more comfortable and better tolerated, largely due to reduced global skin temperature and perceptual thermal strain (Mansfield et al. 2021; Su et al. 2024), even if this comes at the expense of a lower systemic or muscle‑level heat stimulus (Hoekstra et al. 2021). Experts prioritised muscle temperature as a key intervention target, consistent with experimental work demonstrating that local thermal strain is associated with changes in metabolic activity, muscle perfusion, and key anabolic pathways such as HSPs and mTORC1 (Goto et al. 2011; Hafen et al. 2019; Mallette et al. 2019). However, the Delphi findings do not establish muscle temperature as a definitive determinant of neuromuscular or hypertrophic adaptation. Moreover, activation of anabolic signalling pathways (e.g. HSPs, mTORC1) does not necessarily translate into increases in muscle mass or strength, and mechanistic evidence should therefore be interpreted as supportive rather than confirmatory of functional or morphological adaptation.

To contextualise the expert‑derived target muscle temperature within existing experimental evidence, Rodrigues et al. (2020b) demonstrated that vastus lateralis temperature required approximately 80 min to reach ~ 39 °C during localised 42 °C immersion, while core temperature, despite increasing with time, reached its peak below the critical threshold for heat-related risk (~ 39.5–40.0 °C). Importantly, participants in that study did not report the protocol as uncomfortable, indicating that the applied thermal load was both effective and well-tolerated. Nonetheless, Nasir et al. (2026) show that hot-water immersion can cause greater thermal discomfort than other heating methods, suggesting that tolerability may depend on the protocol and the overall thermal load involved. This highlights a potential discrepancy between empirically observed heating kinetics and the expert‑prioritised session duration, such that achieving a 39–40 °C muscle temperature within a 60‑minute session may require higher water temperatures, potentially increasing perceptual strain and reducing comfort.

Although experts prioritised maintaining muscle temperature between 39 and 40 °C, current experimental evidence does not support this range as a strict requirement for neuromuscular or hypertrophic adaptation. Rather, this temperature range should be viewed as a reference point derived from expert judgement that warrants systematic investigation across different heating modalities and populations. Improvements in muscle contractile function and neuromuscular performance have been observed at slightly lower muscle temperatures (≈ 38 °C), particularly following moderate-duration heating protocols (e.g., Hafen et al. 2019; Rodrigues et al. 2020b). These findings suggest that physiological benefits may be achieved within a broader temperature range, likely depending on the heating modality, exposure duration, and targeted outcomes (e.g., strength, hypertrophy, or recovery). Therefore, while the 39–40 °C target remains a useful reference for maximal thermal adaptation, future studies should investigate whether lower temperatures can elicit comparable effects with improved comfort and tolerability.

Although the literature does not define a gold‑standard passive heating intervention for improving muscle strength and mass, experts ranked once‑daily exposure as an important scheduling consideration. This reflects perceived importance rather than established feasibility or adherence, highlighting the need for future studies to evaluate the practicality of such protocols in real‑world settings. While this reflects expert agreement, its implementation in real-world settings may be challenging. For example, the median expert response of 60 min sessions, five days per week, aligns with the upper range of weekly moderate-intensity aerobic physical activity guidelines (Bull et al. 2020). Although passive heating may offer an alternative or adjunct to exercise in specific populations, its translation into practice warrants further investigation, particularly regarding feasibility, accessibility, and user adherence. This is especially important given that time constraints are among the most cited barriers to engaging in physical exercise programs (Matthews et al. 2008; Pagnan et al. 2017).

Empirical studies examining passive heating‑induced changes in muscle strength and mass report divergent outcomes across protocols. For example, longer interventions have produced hypertrophic adaptations, with Goto et al. (2011) demonstrating significant increases in muscle cross-sectional area after ten weeks of thigh heat-and-steam-generating sheet application (8 h per session, 4 days a week). In contrast, Kim et al. (2020b) reported no changes in muscle volume after eight weeks of water-perfused garment heating (90 min, 5 days/week). Short-term interventions have shown benefits in specific contexts: Hafen et al. (2019) attenuated disuse atrophy with short-wave diathermy applied during ten days of immobilisation, while reduced muscle damage markers were observed following a single heat exposure 24 h before eccentric exercise (Nosaka et al. 2007; Saga et al. 2008). These mixed outcomes reflect differences in heating modality, achieved tissue temperature, targeted muscle group, training status, and whether heating was applied as a stand-alone stimulus or in conjunction with other interventions. Recent syntheses caution against over-generalised conclusions that passive heating “works” or “does not work” without careful consideration of FITT-related variables and documentation of endogenous thermal strain (Rodrigues et al. 2025).

In the current Delphi process, experts did not reach consensus on the optimal number of sessions or intervention duration needed for measurable neuromuscular or hypertrophic outcomes. Such a lack of agreement may reflect the variability and limited comparability of available evidence. It also highlights the need for studies that systematically examine dose-response relationships for passive heating, considering both acute and chronic adaptations, across diverse populations and in applied settings. By identifying these parameters, future research could inform more precise recommendations on frequency, duration, and modality to optimise muscular adaptations through passive heating. For best practice and user safety, core temperature monitoring (Round 2: 100%) and cardiovascular strain assessment via blood pressure and heart rate (Round 2: 88.9%) were considered the most important measures to optimise safety during passive heating. In applied settings, evidence from thermal physiology suggests that core temperature above approximately 39.5–40.0 °C substantially increases the risk of heat-related illness (Ioannou et al. 2022; Racinais et al. 2017) and should serve as a threshold for terminating a session. Similarly, practitioners should monitor heart rate responses, with sustained values above approximately 85% of age-predicted maximum heart rate (American College of Sports Medicine 2021), indicating the need to pause or discontinue the intervention, particularly in individuals with cardiovascular risk factors. Blood pressure should be regularly checked, with excessive drops prompting cessation. These thresholds should be adapted for clinical populations and applied alongside symptom monitoring (e.g., dizziness, nausea, disorientation) to ensure safe practice.

Indeed, core temperature plays a vital role in safety during heat exposure (Ioannou et al. 2022). Muscle temperature has been used in the literature as the primary determinant of passive heating during neuromuscular and musculoskeletal interventions. Studies have reported muscle temperature between 37 °C and 39.5 °C (Lloyd et al. 2015, 2017), while others have used core temperature (Brazaitis et al. 2019; Morrison et al. 2004; Thomas et al. 2006) to mimic exercise-like responses (or to compare with exercise). Although it has an important effect, measuring muscle temperature is not always feasible because the necessary measurement expertise for this invasive procedure restricts its use in applied practice. Similarly, accurate measurement of core temperature also poses limitations, as it commonly requires invasive or semi-invasive methods (e.g., rectal or oesophageal probes, or ingestible sensors), which may reduce participant comfort and limit feasibility in applied or community-based interventions. Consequently, although lacking the precision of direct measures, non-invasive surrogates such as skin or tympanic temperature, in conjunction with cardiovascular and subjective indices, are often used to monitor safety and thermal strain during passive heating.

### Intervention target

The lack of consensus regarding a specific target population likely reflects both the limited number of studies examining passive heating effects on muscle strength and mass and the heterogeneity of participant characteristics across existing research. Indeed, the wide range of neuromuscular benefits from passive heating, combined with the lack of a specific population or method, makes it difficult to determine whether a passive heating session should be used differently depending on the intended purpose. However, the experts strongly agreed (Round 2: 87.5%) that the design of passive heating interventions should consider the purpose of passive heating (e.g., rehabilitation to restore function following substantial deconditioning vs. maintenance to limit losses during temporary injury‑related unloading) as well as participant tolerance to heat (e.g., a progressive scaling of temperature to meet the intended target).

Using exercise training principles as a conceptual guide, it would be expected that a passive heating session is individualised. For instance, the session would differ across populations, even when targeting the same outcomes (e.g., applying passive heating to promote rate of torque development improvement in older adults vs. athletes in rehabilitation). However, studies of different responses to the same method across populations do not currently exist. Thus, the dose-response of passive heating to promote muscle strength and muscle mass gains is a novel and interesting area to investigate. As dose-response in exercise can be affected by the physiological factors of each individual, passive heating may also have similarities that could lead to adjustments for the best practice and results. Recent reviews further emphasise that any potential benefits of passive heating in older adults or individuals with cardiovascular or metabolic risk depend on conservative progression, careful screening, and continuous monitoring of physiological strain, particularly when interventions are implemented outside controlled laboratory settings (Rodrigues et al. 2024).

### Intervention safety controls

Inter-individual factors (Round 2: 70%) were considered the most important influences on passive heating outcomes. Training status could affect the outcomes, as most improvements in neuromuscular function were observed in untrained individuals or during or after immobilisation (Hafen et al. 2019), whereas no change occurred in active people. Labidi et al. (2021) reported no increases in muscle strength or muscle mass following six weeks of single‑leg heat therapy using heat pads in healthy, active individuals. Although no study has investigated the effects of diet, sleep and physical activity on heat treatment, the influence of these variables on muscle recovery and adaptation to external load is well established (Burd et al. 2009; Moore and Philp 2020; Yang et al. 2019). Thus, monitoring or manipulating diet and sleep in studies could help elucidate the dose-response relationship between heating and muscle strength and muscle mass outcomes. Experts reached consensus on the importance of screening for contraindications prior to implementing passive heating interventions. This finding highlights safety as a central consideration when designing or studying passive heating protocols rather than constituting clinical practice guidance. Indeed, this should be done thoroughly before prescribing passive heating as a treatment. Although this item has not been extensively developed, specific clinical populations exhibit altered thermoregulatory responses, such as individuals with multiple sclerosis (Davis et al. 2010), diabetes (Kenny et al. 2016), and pregnant (Samuels et al. 2022) or post-menopausal women (Gombert‑Labedens et al. 2025), that must be considered when implementing passive heating interventions. For example, approximately 60–80% of individuals with multiple sclerosis experience symptom exacerbation during heat exposure due to impaired neural control of thermoregulatory effectors and increased heat sensitivity in demyelinated axons (Davis et al. 2010). In people with diabetes, complications such as autonomic neuropathy and vascular dysfunction impair sweat gland function and skin blood flow, reducing heat dissipation and increasing the risk of heat-related illness (Kenny et al. 2016). Pregnant individuals also undergo physiological changes, including increased metabolic heat production and altered cardiovascular function, that affect heat tolerance; however, recent evidence indicates that passive heating (e.g., hot baths or saunas) performed within experimentally defined exposure limits (e.g., duration and environmental conditions) does not raise T_c_ beyond the teratogenic threshold of 39.0 °C, suggesting low risk when appropriately monitored (Ravanelli et al. 2019). These considerations are essential for tailoring safe passive heating protocols to vulnerable populations.

Although the panel did not reach consensus on a single target population, the current evidence suggests that passive heating may be most beneficial for individuals at risk of neuromuscular decline, such as older adults, and for populations unable to engage in traditional resistance training due to injury, illness, or access barriers. Additionally, passive heating shows promise as a supplementary strategy in athletic and rehabilitation settings. These applications are supported by studies demonstrating improvements in muscle mass and strength following heat exposure in inactive or immobilised populations (Hafen et al. 2019; Labidi et al. 2024), while little or no benefit is observed in already active individuals (Labidi et al. 2021; Stadnyk et al. 2018).

### Limitations and future directions

While this study identified areas of expert consensus, the findings reflect informed expert judgement rather than experimental validation. As with all Delphi studies, consensus may evolve as new empirical evidence emerges, and different expert panels may prioritise alternative parameters. Given the novelty and lack of long-term heating intervention studies, future studies are needed to determine the best treatment dose and the target population that would most benefit. Although these results are subjective, consensus-based strategies should be considered for subsequent study design and applied practice. In the absence of formal practice guidelines, these consensus outcomes represent the best available structured guidance to inform research design and early, carefully monitored application, while not substituting for empirically derived recommendations. Furthermore, the reliability of a Delphi exercise is unknown, and it is possible that different outcomes could be achieved if repeated, especially since new experimental data might contradict the opinion presented here.

## Conclusion

This Delphi study identified key considerations for passive heating interventions targeting muscle strength and muscle mass outcomes. First, hot-water immersion was considered the most efficient heating method, with a target muscle temperature of 39 °C to 40 °C maintained for 60 min daily, believed to be most beneficial. More data are required to determine the threshold for positive muscle volume and neuromuscular adaptations induced by passive heating. Secondly, screening users for contraindications and recording body core temperature and cardiovascular strain must be prioritised, and inter-individual factors should be considered in the intervention design to optimise the likelihood of achieving the intended adaptations. Lastly, the development of heating tolerance should be considered in a longitudinal intervention, which could imply adjusting the heating temperature/intensity, to promote the expected benefits. As additional experimental data emerge, expert perspectives on passive heating are likely to evolve. The present findings should therefore be viewed as a consensus‑based framework that identifies priority parameters and safety considerations to guide future research and early translational efforts, rather than definitive practice recommendations.

## Appendix 1

### Round 1 questionnaire–Delphi survey (study 1)

In this first round, a questionnaire with open-ended questions will be provided. You will have 21 days to complete this questionnaire. A reminder will automatically be sent to your email two weeks, one week and three days before the deadline. We appreciate the time you are taking to respond to this questionnaire.

Part1: Participant details.

1. Please provide your primary country of work.
2. Background work experience.
3. What is your H-index? Please choose one of the following:

- < 10
- 11–20
- 21–30
- 31–40
- 40 +
- Rather not say

4. How many peer-reviewed publications do you have on any topic/subject? Please choose one of the following:

- < 50
- 51–100
- 101–200
- 200+

### Part 2: passive heating strategies

Passive heating is thought to promote benefits for neuromuscular function and changes in skeletal muscle mass, including hypertrophy and atrophy prevention. As a result, there is an increasing interest in investigating changes in muscle mass and gains in neuromuscular function using passive heating strategies. However, the optimal passive heating intervention is yet to be identified. For example, it is unclear which heating mode and intensity is most effective. Issues relating to participant safety are also limited. With this in mind, the questions below aim to gather expert opinions that may contribute to a consensus on a passive heating design approach.

Passive heating strategies

When designing a passive heating protocol to improve neuromuscular function and/or muscle changes;

1. What method(s) of passive heating would you consider the most effective for neuromuscular function improvement and/or changes in muscle mass? Please explain.
2. When designing a passive heating protocol to improve neuromuscular function and/or muscle changes; Would you consider that the body surface area exposed (e.g., whole body or part body) to the heat may determine the impact of neuromuscular function and/or muscle mass benefits?
  - (If Yes) How could the body surface area exposed to the heat determine the neuromuscular and/or muscle mass benefits?
3. When designing a passive heating protocol to improve neuromuscular function and/or muscle changes; What intensity (i.e., temperature) do you consider most effective to obtain neuromuscular function improvement and changes in muscle mass?
4. Considering the passive heating methods mentioned above, what thermal strain variable do you use to control the heat stress? E.g., water temperature, air temperature and humidity, muscle temperature, and core temperature.
5. When applying passive heating, what variable do you use to monitor participant safety? E.g., core temperature, heart rate, blood pressure, thermal sensation scales, sweating rate, and skin colour.
6. What is the optimal passive heating session duration (minutes or hours) to obtain neuromuscular function improvement and changes in muscle mass?
7. How frequently (sessions per week) should passive heating be applied to obtain neuromuscular function improvement and changes in muscle mass?
8. Is there an optimal passive heating schedule? E.g., multiple sessions daily, daily, or less regularly?
9. How many sessions or weeks of passive heating intervention are required to achieve beneficial neuromuscular function adaptations?
10. How many sessions or weeks of passive heating intervention are required to achieve muscle hypertrophy or prevent muscle atrophy?
11. Are there any other factors that should be considered regarding the passive heating strategies when designing a protocol to promote neuromuscular function improvement and changes in muscle mass?
  - (If yes) What other factor(s)? Please explain.

Intervention target

12. Specific to neuromuscular function improvement and changes in muscle mass, who do you think would most benefit from passive heating therapies (e.g., sex, age, fitness status, health condition, etc.)? Please explain.
13. Can you please cite specific neuromuscular function adaptations that occur with passive heating intervention?
14. Can you please cite specific musculoskeletal adaptations that occur with passive heating intervention?
15. Please list and explain any other important factors that could interfere with the passive heating protocol, potentially affecting neuromuscular function and muscle mass responses (e.g., environmental conditions, in-session fan use, in-session cold water or ice consumption, hydration status, caffeine consumption or other substances before the session).

Intervention safety controls

16. Please list and explain other factors you consider important when applying heat stress safely.
17. Is there any other variable(s) or aspect(s) you consider important when designing a passive heating protocol that has not been mentioned in this survey?
18. Which other variable(s) or aspect(s) do you consider important that has not been mentioned in this survey?

## Appendix 2

See Table 5

**Table 5:** Consensus level, median and IQR of statements obtained from Round 2 regarding passive heating strategies

Statements		Median rank	% Rank 1 & 2	IQR	Consensus (> = 70%)
1.1.	The most effective heat transfer and thermal conductivity provide a more uniform heating.	1	80%	0	Yes
1.2.	The most accessible for the broadest range of individuals	5	20%	1.75	No
1.3.	The ability to target part- or whole-body regions	3	40%	2	No
1.4.	Heating can occur quickly and be controlled, also increasing pressure.	3.5	20%	1.75	No
1.5.	Prolonged warming effect that promotes a sustained increase in core and muscle temperature.	4	20%	1.75	No
1.6.	It seems to benefit the clinical population, as it is not uncomfortable as other methods.	7	0%	1	No
1.7.	It is efficient, applicable, affordable, and simple to calibrate and maintain.	5.5	20%	3.25	No
2.1.	It is associated with muscle temperature, which impact on neuromuscular and muscle mass responses.	2.5	50%	2	No
2.2.	It influences the heat storage and distribution rate to the body, which may benefit muscle mass.	2	60%	1.75	No
2.3.	It influences the core temperature, which may impair the neuromuscular responses independent of muscle temperature.	4.5	20%	2.75	No
2.4.	Whole-body heating affects core temperature and, thus, oxidative, immune, and cardiovascular stress/strain.	4	40%	2.75	No
2.5.	Local heating is associated with neuromuscular adaptation.	5	20%	1.75	No
2.6.	It may affect the tolerability to the heat, which may impact negatively on the benefits expected.	4.5	10%	2.75	No
3.1.	Muscle temperature	1	60%	2	No
3.2.	Core temperature	2	50%	1	No
3.3.	Water temperature	2	60%	2	No
3.4.	Humidity	7	0%	2	No
3.5.	Ambient temperature	7	0%	2	No
3.6.	Cardiovascular response (e.g., blood pressure, heart rate)	4	0%	3	No
3.7.	Weighed body temperature	8	0%	2	No
3.8.	Skin temperature	6	10%	3	No
3.9.	Thermal comfort and thermal sensation scales	5	0%	1	No
4.1.	Core temperature (rectal or oesophageal)	1	90%	0	Yes
4.2.	Cardiovascular strain (blood pressure, heart rate)	2	80%	0	Yes
4.3.	Thermal sensation	6	0%	2	No
4.4.	Thermal comfort	5	0%	1	No
4.5.	Symptoms and signs: skin colour, speech, nausea, syncope, volitional exhaustion, request for cessation, etc.	3	10%	0	No
4.6.	Temperature of the apparatus	7	0%	3	No
4.7.	Cognitive function/attentiveness/ability to focus forms	7	0%	2	No
4.8.	Skin temperature	8	0%	3	No
4.9.	Subjective ratings	9	0%	0	No
4.10.	Sweating rate	10	0%	1	No
4.11.	Pre-testing is important to avoid low-temperature burns	11	0%	0	No
5.1.	Individual factors (age, sex, body composition)	3	40%	2.75	No
5.2.	Physical fitness (fitness level, training history)	4	40%	3.5	No
5.3.	Health status (e.g., clinical conditions, clinical needs, immobilisation, and underlying disease)	2	60%	1	No
5.4.	The mode of heating (temperature used, equipment available)	4	0%	2.75	No
5.5.	The specific purpose of the intervention (atrophy attenuation vs. maximising or maintaining hypertrophy/strength)	3	50%	3.5	No
5.6.	Core temperature response to the heat	6	0%	1	No
5.7.	Accessibility	7	0%	0.75	No
5.8.	Adherence	8	0%	0	No
5.9.	Supervision	9	0%	0	No
5.10.	Avoiding low-temperature burns	10	10%	0	No
6.1.	Water temperature at 39 °C	5	0%	1.75	No
6.2.	Water temperature at 40 °C	4	20%	1.75	No
6.3.	Water temperature at 41 °C	3	20%	1.75	No
6.4.	Water temperature at 42 °C	5.5	30%	4.25	No
6.5.	Muscle temperature between 30 to 40 °C	2	80%	1	Yes
6.6.	Elevation of 1 to 1.5 °C on core temperature	4.5	10%	2.5	No
6.7.	Elevation of 1 to 1.5 °C on muscle temperature	5.5	40%	4.75	No
7.1.	20–30 min	5	0%	1.75	No
7.2.	45 min	3	30%	2.5	No
7.3.	60 min	2	90%	0.75	Yes
7.4.	90 min	2	70%	2.25	Yes
7.5.	120 min	4	10%	2	No
8.1.	Three sessions per week	3	20%	2	No
8.2.	Four sessions per week	2	50%	3	No
8.3.	Five session per week	2	50%	2	No
8.4.	Six sessions per week	4	40%	2	No
8.5.	Seven sessions per week	5	20%	2	No
9.1.	Once a day	1.50	0.80	1.00	Yes
9.2.	Multiple sessions daily	3.50	0.20	1.00	No
9.3.	Less regularly	2.00	0.80	0.75	Yes

## Appendix 3

See Table 6

**Table 6:** Consensus level, median and IQR of statements obtained from Round 2 regarding intervention target

Statements		Median rank	% Rank 1 & 2	IQR	Consensus (> = 70%)
10.1.	Untrained individuals	4	20%	1.75	No
10.2.	Elderly	3.5	30%	3.25	No
10.3.	Immobilisation (e.g., due to injury)	4	30%	3.5	No
10.4.	Athletes in rehab	5.5	30%	5.5	No
10.5.	Clinical population	4.5	30%	4.25	No
10.6.	People with locomotive problems	6	10%	1	No
10.7.	Anyone can benefit	8	30%	7.75	No
10.8.	Muscle atrophy or weakness aligned with conditions like Parkin’s disease, amyotrophic lateral sclerosis (ALS), Guillain-Barre syndrome, multiple sclerosis, muscular dystrophy, and neuropathy	8	10%	3.5	No
10.9.	Those who have suffered from previous vasodilatory, vasoconstriction issues (i.e., amputees)	9	10%	1	No
10.10.	Returning from or during bed hospitalisation	7	0%	4.75	No
11.1.	Improved peak twitch torque	2	50%	2	No
11.2.	Improved voluntary muscle force or torque	2.5	40%	3.25	No
11.3.	Improved half-relaxation time (faster)	3	20%	1.25	No
11.4.	Improved rate of torque development	2.5	40%	2	No
11.5.	Improved calcium reuptake	5	10%	0.5	No
11.6.	Improved electromechanical delay	6	0%	1.25	No
12.1.	Muscle hypertrophy	2	50%	3	No
12.2.	Changes in mitochondrial function and/or content	3	40%	1.75	No
12.3.	Increased capillarisation	4	40%	2	No
12.4.	Decreased rate of muscle atrophy	2	60%	3	No
12.5.	Heat shock protein expression	3.5	10%	1	No

## Appendix 4

See Table 7

**Table 7:** Consensus level, median and IQR of statements obtained from Round 2 regarding intervention safety control

Statements		Median rank	% Rank 1 & 2	IQR	Consensus (> = 70%)
13.1.	Electric fans and cold-water consumption by helping with heat stress tolerance and comfort	3.5	40%	2.75	No
13.2.	Hydration status by helping with thermoregulatory responses during the session	3.5	20%	2.75	No
13.3.	Inter-individual factors (e.g., trained vs. non-trained, diet, sleep)	2	70%	1.75	Yes
13.4.	Many of the factors listed can improve the potential for gains	7	10%	3.5	No
13.5.	Cooling strategies can negatively affect the benefits of passive heating	8	20%	6.75	No
13.6.	Pharmacological factors may impair the benefits of passive heating	7.5	0%	2.75	No
13.7.	Cooling and heating can co-exist, provided they are done separately and do not interfere with each other’s primary physiological response	8.5	0%	4	No
13.8.	All listed in the question will help on participant comfort	10	0%	3.5	No
13.9.	The phase of the menstrual cycle	10.5	10%	2.5	No
13.10.	Substrate availability (e.g., weight-cutting practices generally have athletes in a nutrient-poor state)	9.5	0%	3.75	No
13.11.	Jetlag (i.e., circadian rhythm offset) can affect the core temperature response	12.5	0%	1.75	No
13.12.	High altitude/hypoxia (people acclimated to high altitude)	13.5	0%	2.5	No
13.13.	Acclimation/acclimatisation status	4.5	30%	2.75	No
14.1.	Monitor participant signs and symptoms during the session	3	40%	2.5	No
14.2.	Having a research/clinician supervising the session	4	30%	3.5	No
14.3.	Monitoring equipment	6	0%	1	No
14.4.	Familiarisation to heat stress	6.5	0%	3.75	No
14.5.	Never leave the patient alone	5	30%	3.75	No
14.6.	Monitoring daily heat stress, external exercise, and heat exposure	8	0%	1	No
14.7.	Emergency care when working with vulnerable people	8.5	20%	2.5	No
14.8.	Screening for contraindications	1.5	70%	1.75	Yes
14.9.	Monitor physiological responses	5	10%	4	No
14.10.	Pre-testing to avoid low-temperature burns	10	0%	0	No
15.1.	Participant tolerance to heat (i.e., increase the intensity of the heat in each session gradually)	2	70%	1	Yes
15.2.	Taking into consideration the purpose of passive heating/intervention (rehab vs. maintenance during injury recovery)	1	70%	1	Yes
15.3.	The financial and environmental cost	5	20%	2	No
15.4.	Find the lowest intensity that promotes benefits to avoid heat radiation	4	0%	2	No
15.5.	The number of measurements could effect participant comfort	4	20%	3	No
15.6.	Participant enjoyment	5	0%	2	No

## Abbreviations

FITT: Frequency, intensity, time, and type
HSPs: Heat shock proteins
IQR: Interquartile range
mTORC1: Mechanistic target of rapamycin complex 1
